# Recurrence and survival after pathologic complete response to preoperative therapy followed by surgery for gastric or gastrooesophageal adenocarcinoma

**DOI:** 10.1038/bjc.2011.175

**Published:** 2011-05-24

**Authors:** R C Fields, V E Strong, M Gönen, K A Goodman, N P Rizk, D P Kelsen, D H Ilson, L H Tang, M F Brennan, D G Coit, M A Shah

**Affiliations:** 1 Gastric and Mixed Tumor Service, Department of Surgery, Memorial Sloan-Kettering Cancer Center, New York, NY 10065, USA; 2 Department of Epidemiology and Biostatistics, Memorial Sloan-Kettering Cancer Center, New York, NY 10065, USA; 3 Department of Radiation Therapy, Memorial Sloan-Kettering Cancer Center, New York, NY 10065, USA; 4 Thoracic Service, Department of Surgery, Memorial Sloan-Kettering Cancer Center, New York, NY 10065, USA; 5 Gastrointestinal Oncology Service, Department of Medicine, Memorial Sloan-Kettering Cancer Center, 1275 York Avenue, Box 314, New York, NY 10065, USA; 6 Gastrointestinal Pathology Service, Department of Pathology, Memorial Sloan-Kettering Cancer Center, New York, NY 10065, USA

**Keywords:** gastric cancer, pathologic complete response, surgery, chemotherapy, radiation therapy

## Abstract

**Background:**

To characterise recurrence patterns and survival following pathologic complete response (pCR) in patients who received preoperative therapy for localised gastric or gastrooesophageal junction (GEJ) adenocarcinoma.

**Methods:**

A retrospective review of a prospective database identified patients with pCR after preoperative chemotherapy for gastric or preoperative chemoradiation for GEJ (Siewert II/III) adenocarcinoma. Recurrence patterns, overall survival, recurrence-free survival, and disease-specific survival were analysed.

**Results:**

From 1985 to 2009, 714 patients received preoperative therapy for localised gastric/GEJ adenocarcinoma, and 609 (85%) underwent a subsequent R0 resection. There were 60 patients (8.4%) with a pCR. Median follow-up was 46 months. Recurrence at 5 years was significantly lower for pCR *vs* non-pCR patients (27% and 51%, respectively, *P*=0.01). The probability of recurrence for patients with pCR was similar to non-pCR patients with pathologic stage I or II disease. Although the overall pattern of local/regional (LR) *vs* distant recurrence was comparable (43% LR *vs* 57% distant) between pCR and non-pCR groups, there was a significantly higher incidence of central nervous system (CNS) first recurrences in pCR patients (36 *vs* 4%, *P*=0.01).

**Conclusion:**

Patients with gastric or GEJ adenocarcinoma who achieve a pCR following preoperative therapy still have a significant risk of recurrence and cancer-specific death following resection. One third of the recurrences in the pCR group were symptomatic CNS recurrences. Increased awareness of the risk of CNS metastases and selective brain imaging in patients who achieve a pCR following preoperative therapy for gastric/GEJ adenocarcinoma is warranted.

Together, gastric and oesophageal adenocarcinoma are the second most common malignancies of the gastrointestinal tract in the United States and worldwide (Kamangar *et al*, 2006; Jemal *et al*, 2010). The US age-adjusted incidence and mortality of gastric/gastrooesophageal junction (GEJ) adenocarcinoma is 7.3 and 5.04 per 100 000 persons, respectively, and the incidence of gastric cancer is rising in the United States in the young age bracket (Anderson *et al*, 2010; Shah and Ajani, 2010). The majority of patients presenting with resectable gastric/GEJ adenocarcinoma will have locally advanced disease (defined as the penetration of the subserosa by the primary tumour (T3), regional nodal involvement (N+), or both (Edge *et al*, 2010)) for which the chance of cure with surgery alone is poor (Hundahl *et al*, 2000; Wu *et al*, 2009; Anderson *et al*, 2010). For these locally advanced cases, several random assignment studies have established additional therapy as the standard of care, including perioperative chemotherapy with or without radiation therapy (RT) (Cunningham *et al*, 2006; Stahl *et al*, 2009), or postoperative chemoradiation (Macdonald *et al*, 2001), highlighting the importance of multidisciplinary care for these patients.

It is well established that pathologic complete response (pCR) following preoperative therapy is associated with improved survival in several malignancies, including breast adenocarcinoma after preoperative chemotherapy±RT (Wolmark *et al*, 2001; Symmans *et al*, 2007; Adams *et al*, 2010; Chavez-Macgregor *et al*, 2010), oesophageal cancer after preoperative chemoradiotherapy (Berger *et al*, 2005; Rohatgi *et al*, 2005; Chao *et al*, 2009; Donahue *et al*, 2009; Park *et al*, 2010), lung cancer after preoperative chemoradiotherapy (Mamon *et al*, 2005; Chen *et al*, 2007), and rectal cancer after preoperative chemoradiotherapy (Maas *et al*, 2010). Notably, the 15–27% rate of pCR after chemoradiotherapy in rectal cancer (Pucciarelli *et al*, 2004) has led some groups to omit surgery and undertake intensive follow-up in select patients who achieve a clinical complete response with no detectable residual tumour after chemoradiotherapy (Habr-Gama *et al*, 2004).

The timing and pattern of recurrence and overall patient survival in patients with gastric/GEJ adenocarcinoma achieving a pCR after preoperative multimodality treatment is not well characterised, with the current description limited to a single series of 24 patients with gastric cancer who achieved a pCR following chemoradiation (Reed *et al*, 2008). Herein, we report our experience in patients with gastric/GEJ adenocarcinoma who received preoperative chemotherapy±RT followed by complete (R0) resection and achieved a pCR. We compare their survival and recurrence to similarly treated patients who did not achieve a pCR.

## Patients and methods

### Patients and pretreatment evaluation

Patients with gastric/GEJ adenocarcinoma who received preoperative therapy were identified from a prospective surgical database at Memorial Sloan-Kettering Cancer Center (MSKCC) from 1985 to 2009. The MSKCC Institutional Review Board approved the study design. Patients with a diagnosis of distal oesophageal carcinoma (Siewert I) were excluded.

Pretreatment evaluation usually consisted of a computed tomography (CT) scan of the abdomen and pelvis, endoscopic ultrasound (EUS), diagnostic laparoscopy with cytologic washings, and selective use of positron emission tomography (PET). We collected patient demographics including age, gender, race, and body mass index; preoperative tumour characteristics including tumour location, pretreatment EUS T-stage, and tumour histology; and preoperative chemotherapy regimen and use of RT.

### Treatment

Preoperative chemotherapy was administered in the outpatient setting and was grouped into: 5-fluorouracil (5-FU) and platinum-based regimens (including epirubicin+cisplatin or oxaliplatin+5-FU or capecitabine (ECF/ECX/EOX/EOF), and cisplatin/5-FU), platinum-based regimens (including cisplatin/CPT11), taxane-based regimens (including taxane+CPT11±5-FU), and other regimens (including 5-FU+doxorubicin+methotrexate (FAMTX), mitomycin C, and doxorubicin).

Preoperative radiation was delivered as multifield, external-beam megavoltage radiation using high-energy linear accelerators (6 or 15 MV). Treatment generally included five daily fractions of 1.8 Gy per week over a 5.5-week course with a total radiation dose of 50.4 Gy. The superior field border extended ∼5 cm cranial to the tumour, and the inferior border extended caudally to include the coeliac lymph node (LN) region. The anterior, posterior, and lateral field borders were ∼2 cm beyond the tumour, as defined by pretreatment imaging. The locoregional LNs were included in the radiation field.

After preoperative treatment, patients underwent gastrectomy or oesophagogastrectomy with two-field (for oesophagogastrectomy) or D2 (for gastrectomy) lymphadenectomy and splenic preservation whenever possible. A curative (R0) resection was defined as the removal of all visible disease and associated nodal basins with negative microscopic surgical margins on final pathologic review. Pathologic staging is reported according to the American Joint Committee on Cancer staging guidelines (7th edn) for gastric adenocarcinoma (Edge *et al*, 2010). Surgical treatment characteristics collected included operative and pathologic details, including extent of gastrectomy/oesophagogastrectomy, type of LN dissection, and resected specimen pathologic analysis (T-stage, N-stage, number of LN examined, and pathologic treatment effect).

Follow-up laboratory and imaging studies and additional postoperative treatment were at the discretion of the treating physician(s) or as directed by a patient-enrolled protocol (Kelsen *et al*, 1992, 1997; Bains *et al*, 2002; Brenner *et al*, 2004, 2006; Anderson *et al*, 2007; Schwartz *et al*, 2009). Patients were generally followed every 2–3 months for the first 2 years, followed by every 6–12 months thereafter. Recurrence was confirmed radiographically and/or pathologically and described as local/regional (including peritoneal) or distant. Date of recurrence was defined as the first notation in the medical record indicating the recurrence. Disease status at last follow-up and cause of death were determined by the medical record, death certificates, and follow-up correspondence.

### Pathology

Pathologic complete response was defined as fibrosis or fibroinflammation within an entirely submitted and evaluated gross lesion without microscopic evidence of carcinoma, and histologically negative nodes. Non-pCR was defined as any evidence of viable carcinoma, either at the primary site or in the resected regional LN. The pathologic stage of residual carcinoma in the non-pCR group was based on the deepest focus of viable malignant epithelium in the gastric and oesophageal wall and/or any carcinoma found in the LN analysis. Pathologic treatment effect was analysed and quantified on a graded, per cent scale as previously described (Mansour *et al*, 2007). Positive LNs were defined as the presence of any viable tumour cells within LNs.

### Statistical analysis

Statistical analysis was performed using the R package, version 2.10 (http://www.r-project.org). Patient, tumour, and treatment variables were compared between the pCR and non-pCR groups using the *χ*^2^ and Wilcoxon rank sum test with continuity correction for categorical and continuous variables, respectively. Recurrence-free survival (RFS) was compared between the pCR and non-pCR groups using the log-rank test. Recurrence location was compared using the *χ*^2^ test. Kaplan–Meier methods were used to estimate overall survival (OS), disease-specific survival (DSS), and RFS probability between pCR and non-pCR groups and compared using the log-rank test (Kaplan and Meier, 1958). Death without a recurrence was considered as a competing cause of failure (Prentice *et al*, 1978; Satagopan *et al*, 2004). Estimated cumulative incidence of recurrence was performed using the subdistribution method and compared using Gray's test (Gray, 1988).

## Results

**Figure 1:**
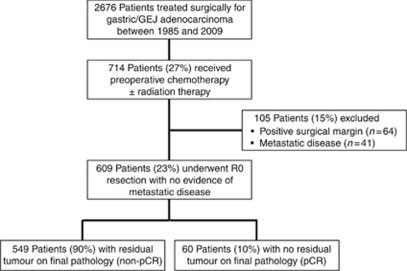
Patient study group CONSORT diagram.

From 1985 to 2009, 2676 patients underwent surgical treatment for gastric or GEJ (Siewert II/III) adenocarcinoma at MKSCC. In all, 714 of these patients (27%) received preoperative chemotherapy±RT. One hundred and five patients (15%) had either positive surgical margins after resection (64 patients, 9%) or presence of metastatic disease at surgical exploration/resection (41 patients, 6%) and were excluded from subsequent analysis. The final study population was 609 patients with gastric/GEJ adenocarcinoma treated with preoperative therapy (280 (46%) chemoradiotherapy, 329 (54%) chemotherapy alone) followed by complete (R0) resection. Sixty patients (8.4% of all preoperative treatment patients; 10% of preoperative treatment patients who underwent R0 resection) demonstrated no residual tumour on final pathology and are defined as the pCR group, and the remainder (*n*=549, 90% of all preoperative treatment patients who underwent R0 resection) had residual evidence of malignancy and are defined as the non-pCR group (Figure 1).

Table 1 lists the patient characteristics of the pCR and non-pCR patients. There were no differences between the pCR and non-pCR groups with respect to patient age, race, pretreatment EUS T-stage, or histology (Lauren or differentiation). Most patients had advanced T-stage tumours (⩾80% T3 for both pCR and non-pCR patients). Forty-seven patients who received chemoradiation (17%) achieved a pCR, and 12 (26%) of these patients recurred. Thirteen patients who received chemotherapy alone (4%) achieved a pCR, and 2 (17%) of these patients recurred. Patients who received taxane-based therapy more commonly also received concurrent radiotherapy and therefore more commonly achieved a pCR.

Table 2 lists the pathologic T- and N-stages and extent of surgical nodal resection. There were no differences in the extent of LN dissection (D1, D2, or D3) between the pCR and non-pCR groups. The non-pCR patients had a mean of 3.0 (range 1–8) positive LNs and a mean pathologic treatment effect of 45% (range 10–95). The pCR group had a higher mean number of LNs examined (29 *vs* 23), *P*=0.04.

**Figure 2:**
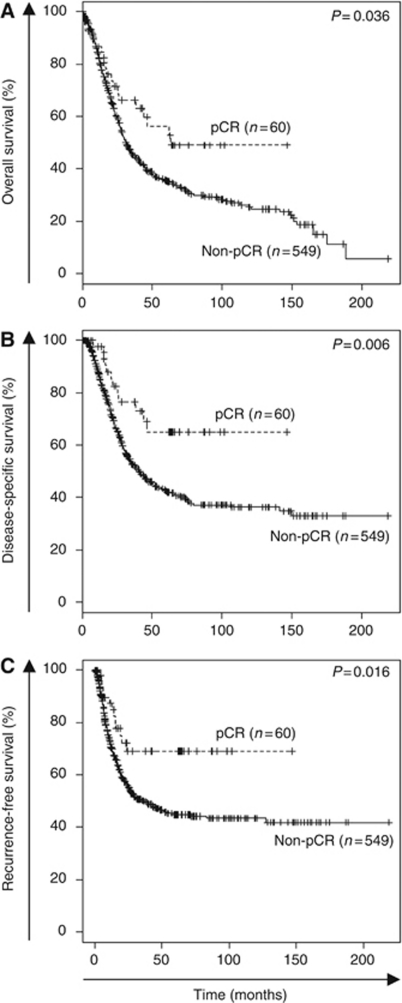
Kaplan–Meier estimates of overall (**A**), disease-specific (**B**), and recurrence-free (**C**) survival stratified by pathologic complete response (pCR) *vs* non-pathologic complete response (non-pCR). Statistical comparisons between pCR and non-pCR groups were determined using the log-rank test.

Median follow-up for all surviving patients was 46 months (interquartile range=16–90). Of the 549 patients with a non-pCR, 153 (28%) received postoperative (adjuvant) chemotherapy, and of the 60 patients with a pCR, one patient received additional postoperative chemotherapy. Overall survival, DSS, and RFS was significantly greater in the pCR group compared with the non-pCR group (Figure 2). The timing and pattern of recurrences are summarised in Table 3. For patients achieving a pCR, there was no difference in recurrence between patients who received chemoradiotherapy *vs* chemotherapy alone (26% and 15%, respectively, *P*=0.2). While the non-pCR group had a higher risk of recurrence at 1, 3, and 5 years (5-year recurrence=27 *vs* 51% for pCR and non-pCR, respectively, *P*=0.02), the pattern of recurrence was similar. There was no difference in the distribution of local/regional *vs* distant recurrences (43% *vs* 57%, respectively) between pCR and non-pCR groups. However, we did observe a significantly higher incidence of first recurrences in the central nervous system (CNS) in pCR (36%) compared with non-pCR (4%) patients (*P*=0.01). All of the patients with a CNS recurrence in the pCR group presented symptomatically (four with seizures and one with localising neurologic symptoms), and similarly 8 of the 10 patients (4%) in the non-pCR group who initially recurred in the CNS presented symptomatically (seven with seizures and one with localising neurologic symptoms).

**Figure 3:**
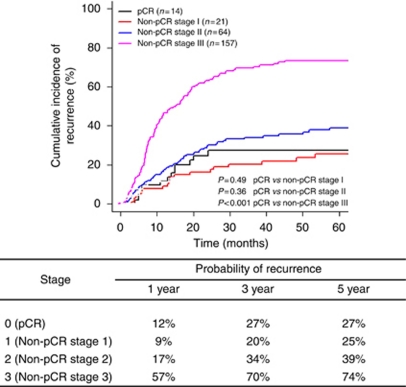
Cumulative incidences and probabilities of recurrence by stage (treating death from other causes as a competing risk) in patients undergoing preoperative chemotherapy±radiation therapy for gastric and gastrooesophageal junction adenocarcinoma, followed by R0 resection. Abbreviations: pCR=pathologic complete response, non-pCR=non-pathologic complete response.

Figure 3 summarises the probability of recurrence by final pathologic stage when treating death from other causes as a competing risk. When compared with pCR patients, the probability of recurrence is significantly higher only for pathologically stage III (pIII) non-pCR patients (5-year CI of recurrence=74 *vs* 27%, *P*<0.001). Among the pIII patients, although the majority were stage III by virtue of residual nodal involvement, the three node negative (i.e., T4N0) pIII patients also had a high risk of recurrence, each one developing recurrence within 1 year of resection. There is no significant difference in the probability of recurrence between pCR patients and stage pI or stage pII non-pCR patients (5-year CI of recurrence=39% and 25%, respectively, *P*=0.49 for pCR *vs* non-pCR stage I and *P*=0.36 for pCR *vs* non-pCR stage II).

Table 4 provides clinical characteristics of those patients with pCR who developed recurrence (*n*=14, 23%). Five patients (36% of pCR recurrences; 8% of all pCR patients) developed CNS recurrence as their first site of recurrence, with a mean time to recurrence of 12.6±7.7 months (range 5–24 months). Treatment after CNS recurrence consisted of whole brain RT in two patients, surgery (craniotomy) in one patient, and no treatment in two patients. All five pCR patients with CNS recurrences died of their disease, with a mean time from recurrence to death of 9.6 months (range=2–26 months). Of note, 6 of the 14 pCR patients that recurred (43%) had local/regional recurrence (anastomotic or regional nodal), all of whom received preoperative chemoradiation.

## Discussion

In the past 10 years, results of several randomised controlled trials have established multimodality therapy as the standard of care for locally advanced gastric/GEJ adenocarcinoma (Macdonald *et al*, 2001; Cunningham *et al*, 2006; Stahl *et al*, 2009; Schuhmacher *et al*, 2010). Reflective of the multidisciplinary approach to locally advanced gastric and GEJ adenocarcinoma, we describe the outcome of patients who received preoperative chemotherapy or chemoradiation followed by complete surgical resection and who achieved a pCR to preoperative therapy. We found a 10% pCR rate in patients with gastric/GEJ adenocarcinoma treated with preoperative chemotherapy±RT followed by R0 resection (17% with prior chemoradiation and 4% with chemotherapy alone). Importantly, despite achieving a pCR with preoperative therapy and independent of type of therapy, the risk of recurrence remains significant; indistinguishable from patients who were downstaged to pathologic stage I or II following preoperative therapy. There is a substantial rate of CNS first recurrences (8% of all pCR patients and 36% of the pCR patients who developed a recurrence) in this cohort of patients, with each CNS recurrence presenting with life-threatening neurologic symptoms.

The biology of tumours that completely regress with preoperative therapy is likely to be distinct from tumours that did not achieve a pCR (Ajani, 2005; Berger *et al*, 2005) and is reflected in RFS and OS. As demonstrated in other malignancies (Wolmark *et al*, 2001; Berger *et al*, 2005; Mamon *et al*, 2005; Rohatgi *et al*, 2005; Chen *et al*, 2007; Chao *et al*, 2009; Donahue *et al*, 2009; Adams *et al*, 2010; Maas *et al*, 2010; Park *et al*, 2010), patients with gastric/GEJ adenocarcinoma who achieve a pCR following preoperative therapy have significant improvements in 5-year OS (60 *vs* 35%), DSS (67 *vs* 43%), and RFS (69 *vs* 45%) when compared with the group who did not achieve a pCR. However, despite achieving a pCR, we noted a significant risk of recurrence in this cohort of patients. Specifically, as shown in Figure 3, there are no differences in the probability of recurrence between the pCR and posttreatment stage I and II patients. The distribution of local/regional (43%) *vs* distant recurrence (57%) in the pCR and non-pCR groups is identical. However, there is a significantly higher rate of CNS first recurrences in the pCR (36%) compared with the non-pCR (4%) cohort. The increased risk of developing CNS metastases in patients achieving a pCR is likely due to diminished penetration of the CNS by all of the chemotherapeutic agents in the treatment of gastric/GEJ cancer (Chabner and Longo, 2011). Non-pCR patients, in contrast, are more likely to have persistent micrometastatic disease in systemic circulation, and are therefore more likely to have a non-CNS site of first recurrence. Although CNS recurrences may be more prevalent in patients with prolonged survival, we would highlight that in our cohort, three of the five CNS recurrences in the pCR group developed recurrence early (i.e., <13 months) in their postoperative period, making our findings more noteworthy. It is well established that CNS metastases occur in ∼50% of patients with locally advanced non-small-cell lung cancer (NSCLC) (Mamon *et al*, 2005). In patients with NSCLC treated with preoperative chemoradiation that have a pCR at the time of resection, there remains a 43% rate of CNS metastases as the site of first failure, which represents 71% of all isolated recurrences (Chen *et al*, 2007). This observation has led to the use of prophylactic cranial irradiation in patients with stage III NSCLC treated with preoperative chemoradiotherapy and curative surgery, a strategy that has significantly reduced the risk of CNS metastases (18.0 *vs* 7.7%, unadjusted odds ratio=2.52, *P*=0.004). However, this strategy has not improved OS or DFS (Gore *et al*, 2011). The rate of CNS recurrence in NSCLC is substantially higher than the 8% rate of CNS as the site of first failure in patients with gastric/GEJ adenocarcinoma who achieved a pCR following preoperative therapy, suggesting a limited value of prophylactic whole brain radiation in this select population.

Interestingly, patients with a pCR had higher numbers of LNs examined in the pathologic specimen when compared with non-pCR patients (29 *vs* 23). In rectal cancer, it is suggested that interactions between tumour and host immune cells may be different between pCR and non-pCR tumours (Ogino *et al*, 2010). Increased LN count, and in particular increased *negative* LN count, has been found to be associated with increased survival in colorectal cancer (Chang *et al*, 2007). Patients who achieve a pCR may elicit a stronger immune response, resulting in more numerous and larger regional LN, suggesting a possible biologic/immunologic difference in the host response to these tumours (Johnson *et al*, 2006; Ogino *et al*, 2010). The low frequency of pCR and varied overall histologic response rates to preoperative therapy highlight the importance of ongoing research to identify response to therapy early. We and others have examined FDG-PET/CT for this purpose (Lordick *et al*, 2007; Shah, 2007; Ott *et al*, 2008; Wieder and Weber, 2009). A presently accruing study at our institution is studying the ability of FDG-PET to discriminate responders *vs* non-responders to preoperative chemotherapy for locally advanced gastric cancer and to salvage non-responding patients with alternate chemotherapy (Shah, 2011).

This retrospective evaluation reflects the current multidisciplinary approach to patients with gastric cancer in which proximal gastric tumours (Siewert type II or III) may receive either chemotherapy alone or combined modality chemoradiation before surgical resection. Our data are not intended to compare and contrast the merits of these two distinct treatment approaches, but rather are focused on the risk and pattern of recurrence of patients who achieve a significant and complete pathologic response to preoperative therapy. Notably, we did not observe a difference in recurrence rate between those receiving chemoradiotherapy (26%) and those receiving chemotherapy alone (15%). This may, in part, be due to the low overall rate of pCR to chemotherapy alone (4%), corresponding to our limited statistical power to compare these two groups.

We acknowledge that our observations are based on a small number of total events (i.e., 14 recurrences in 60 pCR patients, with 5 CNS recurrences). However, to our knowledge this represents the largest reported series describing patients with a pCR after preoperative treatment and surgical resection in gastric/GEJ adenocarcinoma. All of the patients with CNS metastases presented with symptomatic seizures or neurologic symptoms. Early detection of brain metastases may identify these patients before they experience seizures or symptoms and allow for early treatment (stereotactic RT and/or surgery). These data support having an increased awareness of the risk of the CNS as the first site of recurrence in this cohort of patients. Considering that all CNS metastases developed within 2 years of follow-up (range 5–24 months), selective surveillance brain imaging (contrast enhanced CT or MRI) to identify CNS disease before the onset of symptoms during the first 2 years of follow-up would be reasonable. Additionally, we noted that despite achieving a pCR, there was a 43% incidence of local/regional recurrence. Four patients (7% of all pCR patients and 29% of all pCR recurrences) developed a local recurrence as the site of first recurrence. Thus, pCR does not obviate the need for continued local–regional surveillance of this patient cohort.

In summary, pCR following preoperative chemotherapy±RT followed by surgical resection for gastric/GEJ adenocarcinoma occurs in a minority of patients. When compared with non-pCR patients, a pCR results in improved survival; however, there remains a significant rate of recurrence. Patients that achieve a pCR after preoperative therapy have a similar risk of recurrence to posttreatment pathological stage I and II tumours. In addition, there is a significantly higher incidence of symptomatic CNS first recurrences in pCR patients. These findings have important clinical implications: care providers should be cognizant of the risk of symptomatic CNS recurrences in this select cohort of patients and should consider selective brain imaging for early identification and treatment of CNS metastases.

## Figures and Tables

**Table 1:** Patient, tumour, and preoperative treatment variables in patients undergoing preoperative chemotherapy±radiation therapy for gastric and gastrooesophageal junction adenocarcinoma, followed by R0 resection

	**Number (%)**		
**Variable**	**Non-pCR patients (*n*=549)**	**pCR patients (*n*=60)**	***P*-value^a^**
Age (median, IQR)	62 (54–69)	64 (56–69)	0.18^b^
Preoperative BMI (median, IQR)	27 (24–30)	28 (25–32)	**0.04^b^**
			
*Gender*			
Male	403 (73)	52 (87)	**0.02**
Female	146 (27)	8 (13)	
			
*Race*			
Caucasian	487 (89)	57 (95)	
African-American	24 (4)	1 (2)	0.64
Asian/Pacific Islander	31 (6)	2 (3)	
Other/unknown	7 (1)	0 (0)	
			
*Tumour location*			
GEJ	312 (57)	47 (78)	
Gastric, proximal	74 (13)	6 (10)	
Gastric, body	71 (13)	4 (7)	**0.02**
Gastric, distal	85 (15)	3 (5)	
Gastric, diffuse	7 (1)	0 (0)	
			
*Pretreatment EUS T-stage*			
T1	4 (1)	0 (0)	
T2	72 (13)	10 (17)	
T3	447 (81)	50 (83)	0.43
T4	15 (3)	0 (0)	
Not performed	11 (2)	0 (0)	
			
*Histology – Lauren classification*			
Diffuse	169 (31)	19 (32)	
Intestinal	333 (61)	39 (65)	0.62
Mixed	47 (9)	2 (3)	
			
*Histology – differentiation*			
Well	12 (2)	3 (5)	
Moderate	207 (38)	14 (23)	0.25
Poor	330 (60)	43 (72)	
			
*Preoperative chemotherapy regimen*			
5-FU and platinum based	221 (39)	23 (34)	
Platinum based	157 (27)	15 (22)	**0.0007**
Taxane based	130 (23)	28 (41)	
Other	65 (11)	2 (3)	
			
*Preoperative radiation treatment*			
Yes	233 (42)	47 (78)	**<0.0001**

Abbreviations: 5-FU=5-fluorouracil; BMI=body mass index; GEJ=gastrooesophageal junction; IQR=interquartile range; pCR=pathologic complete response; EUS=endoscopic ultrasound.

a *χ*^2^ text, except where otherwise noted by ‘b’.

b Wilcoxon rank sum test with continuity correction. Bold values indicate significant differences.

**Table 2:** Pathologic variables in patients undergoing preoperative chemotherapy±radiation therapy for gastric and gastrooesophageal junction adenocarcinoma, followed by R0 resection

	**Number (%)**		
**Variable**	**Non-pCR patients (*n*=549)**	**pCR patients (*n*=60)**	***P*-value**
*pT stage* a			
T0^b^	11 (2)	60 (100)	
T1a	21 (4)	0	
T1b	48 (9)	0	
T2	125 (23)	0	N/A
T3	243 (44)	0	
T4a	93 (17)	0	
T4b	8 (1)	0	
			
*pN stage* a			
N0	241 (44)	60 (100)	
N1	131 (24)	0	
N2	97 (18)	0	N/A
N3a	59 (11)	0	
N3b	21 (4)	0	
			
*Extent of surgical lymphadenectomy*			
D1	20 (4)	1 (2)	
D2	513 (93)	57 (95)	0.72^c^
D3	16 (3)	2 (3)	
Number of positive LN (for ⩾N1; mean, IQR)	3.0 (1–8)	0 (0)	N/A
Number of LN examined (mean, IQR)	23 (15–29)	29 (25–32)	**0.04^d^**
Pathologic treatment effect (mean %, IQR)	45 (10–95)	100	N/A

Abbreviations: IQR=interquartile range; LN=lymph node; N/A=not applicable (no statistical analysis performed, as differences are defined by subgroup stratification); pCR=pathologic complete response.

a AJCC 7th edn, 2010.

b T0N1 (*n*=9) and T0N2 (*n*=2) – 1 patient has recurred after a median follow-up of 17 months.

c *χ*^2^ text.

d Wilcoxon rank sum test with continuity correction. Bold values indicate significant differences.

**Table 3:** Timing and patterns of recurrence in patients undergoing preoperative chemotherapy±radiation therapy for gastric and gastrooesophageal junction adenocarcinoma, followed by R0 resection

	**Number (%)**		
**Variable**	**Non-pCR patients (*n*=549)**	**pCR patients (*n*=60)**	***P*-value**
*Recurrence*			
Yes	242 (44)	14 (23)	—
			
*Probability of recurrence*			
1 year	28%	12%	
3 years	46%	27%	**0.01^a^**
5 years	51%	27%	
			
*First recurrence location* b			
Local/regional	95 (43)	6 (43)	1.00^c^
Distant	128 (57)	8 (57)	
CNS as site of first recurrence	10 (4)	5 (36)	**0.01^c^**

Abbreviations: CNS=central nervous system; pCR=pathologic complete response.

a Log-rank test.

b Recurrence location information available in 223 out of 242 patients (92%) that recurred in the non-pCR patient group.

c *χ*^2^ test. Bold values indicate significant differences.

**Table 4:** Location and timing of first recurrence in 14 of 60 (23%) patients following a pathologic complete response to neoadjuvant chemotherapy±radiation therapy for gastric or gastrooesophageal junction adenocarcinoma

**Recurrence location**	**Time to recurrence (months)**	**Treatment**	**Status at last follow-up**	**Time from recurrence to death or last follow-up (months)**
CNS	15	None	DOD	1
CNS^a^	5	Whole brain RT	DOD	17
CNS	13	Surgery	DOD	26
CNS	6	Whole brain RT	DOD	2
CNS, liver^a^	24	None	DOD	2
Liver	5	Platinum/CPT11	DOD	12
Lung	54	Platinum/Taxol	AWD	38
Skin	4	FOLFIRI	DOD	16
Regional LN	19	Platinum/Taxol	AWD	27
Regional LN	11	Taxol	DOD	36
Local	20	Taxol	DOD	24
Local	14	Surgery	DOD	1
Local	6	Taxol/CPT11	DOD	11
Local	15	Taxol/CPT11	DOD	10

Abbreviations: AWD=alive with disease; DOD=died of disease; CNS=central nervous system; FOLFIRI=5-FU+leucovorin+irinotecan; LN=lymph node; RT=radiation therapy.

a Denotes gastric primary.
